# Tackling barriers to scale up human papillomavirus vaccination in China: progress and the way forward

**DOI:** 10.1186/s40249-023-01136-6

**Published:** 2023-09-21

**Authors:** Xue-Lian Zhao, Shang-Ying Hu, Jia-Wei Hu, Hong-Hao Wang, Tian-Meng Wen, Yu-Shu Feng, You-Lin Qiao, Fang-Hui Zhao, Yong Zhang

**Affiliations:** 1 Department of Epidemiology, National Cancer Center/National Clinical Research Center for Cancer/Cancer Hospital, Chinese Academy of Medical Sciences and Peking Union Medical College, 17 South Panjiayuan Lane, P.O. Box 2258, Beijing, 100021 China; 2 School of Population Medicine and Public Health, Chinese Academy of Medical Sciences and Peking Union Medical College, Beijing, 100730 China; 3 Teaching and Research Section of Epidemiology, National Cancer Center/National Clinical Research Center for Cancer/Cancer Hospital, Chinese Academy of Medical Sciences and Peking Union Medical College, 17 South Panjiayuan Lane, P.O. Box 2258, Beijing, 100021 China

**Keywords:** Human papillomavirus, Vaccination, Progress, Challenge, China

## Abstract

The human papillomavirus (HPV) vaccine is the first vaccine developed specifically targeting the prevention of cervical cancer. For more than 15 years, China has expedited a series of efforts on research and development of the domestically manufactured HPV vaccines, producing local population-based evidence, promoting free HPV vaccination from pilots, and launching action plans to tackle barriers in the scale-up of HPV vaccination. To further roll out the HPV vaccination program in China, several challenges should be addressed to support the steps forward. The availability of more locally manufactured HPV vaccines, pricing negotiation and local evidence supporting the efficacy of one-dose schedule would greatly alleviate the continued supply and financial constraints in China. Meanwhile, more attention should be paid to girls living in low-resource areas and males to ensure equal access to the HPV vaccination. Furthermore, linkage to secondary prevention and further real-world monitoring and evaluation are warranted to inform effective cervical cancer prevention strategies in the post-vaccine era.

**Graphical Abstract:**

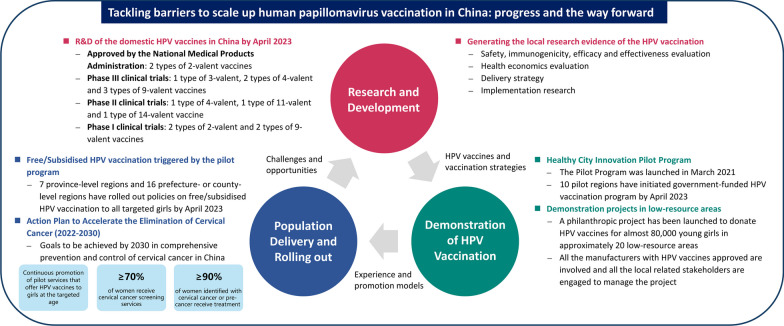

**Supplementary Information:**

The online version contains supplementary material available at 10.1186/s40249-023-01136-6.

## Background

Cancer is a leading cause of death worldwide, accounting for nearly 10 million deaths in 2020 [1]. In 2006, the world’s first vaccine that can prevent cancer—the prophylactic human papillomavirus (HPV) vaccine was approved to protect women from cervical cancer. With the HPV vaccine, cervical cancer is expected to become the first malignant tumor to be eliminated. Accordingly, the World Health Organization (WHO) launched a global strategy to accelerate cervical cancer elimination in 2020, meeting the following targets by 2030 as three powerful weapons: 90% of girls fully vaccinated with the HPV vaccine by 15 years of age, 70% of women screened using a high-performance test by age 35 and again by 45, and 90% of women identified with the cervical disease treated [2]. As of April 2023, six prophylactic HPV vaccines have been approved worldwide, and 134 (69%) of the 194 WHO member states have introduced HPV vaccination in the national immunization program (NIP) [3]. However, global coverage of the first dose of HPV vaccination by age 15 in females is estimated at 21% in 2022, far behind the 2030 elimination target of 90% [4]. In the future, attention should be paid to ensure more countries nationally introduce the HPV vaccine into their immunization programs, especially for countries with high cervical cancer burden, to further improve program performance globally.

China accounts for about a fifth of the global burden of cervical cancer, with about 109,741 new cases of cervical cancer in 2020 [1]. But the availability of the first HPV vaccine in Chinese mainland has been delayed by almost 10 years, resulting in approximately 114 million 9–14 years old girls missing out on the best opportunity to protect them from cervical cancers and precancers [5, 6]. We predict that 783,000 cervical cancer cases and 435,000 related deaths could be preventable in this population by implementing a national HPV vaccination program targeting 9–14 years old girls (90% vaccination coverage with bivalent HPV vaccine), between 2006 and 2015 [6, 7]. However, China has not yet included the HPV vaccine in the national immunization program (NIP). It is estimated that during 2022–2030, each year of delay in the initiation of the NIP could result in the occurrence of more than 119 thousand new cases and 42 thousand deaths of cervical cancer [8]. To compensate for the costly delay and to accelerate the full roll-out of the HPV vaccination program, China has made aggressive efforts to develop domestic HPV vaccines, provide local evidence, explore tailored strategies, initiate free vaccination, and launch a national action plan for steps forward. Here, we summarize the progress made in promoting HPV vaccination in China and propose challenges and suggestions for further scaling up HPV vaccination in the national program.

### Accelerating research and development (R&D) of the domestically manufactured HPV vaccine and evidence accumulation

China is a latecomer in marketing the first HPV vaccine, but it takes its place in the front ranks of the world for R&D of the HPV vaccines. In 2016–2018, three types of imported HPV vaccines were approved successively, which made HPV vaccines available in Chinese mainland [5]. However, the high price with the cost of per-dose regimen ranging from USD 80 to USD 180 and the insufficient supply of the imported HPV vaccine has made it poorly accessible to Chinese women and prevented its widespread use in China [9]. Heralded as a huge step forward, the first domestically manufactured HPV vaccine (Cecolin™) was approved in China in December 2019 and prequalified by WHO in October 2021 [10, 11]. Hereafter, China has become the third country capable of producing HPV vaccines worldwide. Meanwhile, the high production efficiency and low cost of the *Escherichia coli*-based manufacturing process of the Cecolin™ is a promising improvement with regard to accessibility for other resource-limited countries. In 2022, the second domestically manufactured HPV vaccine (Walrinvax™) was also approved in China [5]. To date, China has approved five types of HPV vaccines against cervical cancer, making it the country with the most types of HPV vaccines. Presently, at least 13 types of HPV vaccines are undergoing clinical trials in China, and more products are in the pipeline to be approved in years ahead [5] (Table 1). The rapid development of the Chinese HPV vaccines could provide more possibilities to alleviate the supply and financial constraints of HPV vaccines in China. For the world, the Cecolin™ that has been prequalified by the WHO allows it to be procured through agencies such as the United Nations International Children's Emergency Fund (UNICEF), thereby supporting the global aggregate demand for the HPV vaccines. Meanwhile, Walrinvax™ is currently undergoing the WHO prequalification review and is anticipated to be involved for global use shortly afterward.

**Table 1:** Prophylactic HPV vaccine development in China, 2023

Manufacturer	Vaccine	Expression system	Current phase of R&D
GSK	HPV-2 (16, 18)	Insect cell	Approved in 2016
Merck	HPV-4 (6,11,16,18)	Yeast (*Saccharomyces cerevisiae*)	Approved in 2017
Merck*	HPV-9 (6,11,16,18,31,33,45,52,58)	Yeast (*Saccharomyces cerevisiae*)	Approved in 2018
Xiamen Innovax	HPV-2 (16,18)	*Escherichia coli*	Approved in 2019
Shanghai Zerun (Walvax)	HPV-2 (16,18)	Yeast (*Pichia pastoris*)	Approved in 2022
CNBG/CDIBP (Chengdu/Beijing)	HPV-4 (6,11,16,18)	Yeast (*Hansenula polymorpha*)	Phase III
Shanghai Bovax	HPV-4 (6,11,16,18)	Yeast (*Hansenula polymorpha*)	Phase III
Shanghai Bovax	HPV-9 (6,11,16,18,31,33,45,52,58)	Yeast (*Hansenula polymorpha*)	Phase III
Xiamen Innovax	HPV-9 (6,11,16,18,31,33,45,52,58)	*Escherichia coli*	Phase III
Beijing Health Guard	HPV-3 (16,18, 58)	*Escherichia coli*	Phase III
Beijing Health Guard	HPV-9 (6,11,16,18,31,33,45,52,58)	*Escherichia coli*	Phase III
CNBG/SIBP (Shanghai)	HPV-4 (16,18,52,58)	Yeast (*Pichia pastoris*)	Phase II
CNBG/CDIBP (Chengdu/Beijing)	HPV-11 (6,11,16,18,31,33,45,52,58,59,62)	Yeast (*Hansenula polymorpha*)	Phase II
Beijing Nuoning / Beijing SinoCellTech	HPV-14 (6,11,16,18,31,33,35,39,45,51,52,56,58,59)	Insect cell	Phase II
Jiangsu RecBio	HPV-9 (6,11,16,18,31,33,45,52,58)	Yeast (*Hansenula polymorpha*)	Phase I
Jiangsu RecBio	HPV-2 (16,18)	Yeast (*Hansenula polymorpha*)	Phase I
Shanghai Zerun (Walvax)	HPV-9 (6,11,16,18,31,33,45,52,58)	Yeast (*Pichia pastoris*)	Phase I
Jiangsu RecBio	HPV-2 (6,11)	Yeast (*Hansenula polymorpha*)	Phase I

*BLA* Biologics License Application; *GSK* GlaxoSmithKline; *HPV* Human Papillomavirus; *CNBG* China National Biotec Group; *CDIBP* Chengdu Institute of Biological Products; *SIBP* Shanghai Institute of Biological Products, *R&D* Research and Development

*Conditionally approved by NMPA in 2018, the phase III clinical trial was initiated in June 2019 in China

Local population-based research evidence is essential for the approval of HPV vaccines in China. For more than 15 years, Chinese scientists have been committed to generating high-quality scientific evidence from local populations on the safety, immunogenicity, and efficacy of HPV vaccines to accelerate their approval and implementation [10, 12–16]. The latest research evidence indicated that both the imported bivalent (Cervarix™) and quadrivalent HPV vaccine (Gardasil™) showed an acceptable safety profile and a long-term protective effect (8-year and 10-year follow-up, respectively) against HPV-related precancerous diseases among Chinese women. The first domestic vaccine (Cecolin™), which protects against two HPV strains, is 97.3% effective against persistent infection, and 100.0% effective against HPV 16 and 18-associated cervical intraepithelial neoplasia grade 2 or higher (CIN2 +), vulvar intraepithelial neoplasia grade 2 or higher (VIN2 +), or vaginal intraepithelial neoplasia grade 2 or higher (VaIN2 +) for up to 5.5 years of follow-up, on par with its international alternatives [14].

When introducing HPV vaccine into the national program, all the HPV vaccines are cost-effective in China among the WHO-recommended primary target group of females at 9–14 years with the incremental cost-effectiveness ratio (ICER) less than the gross domestic product (GDP) per capita for 2021 (USD 12,457.85) [assuming a lower tender price than the market price based on the Pan American Health Organization Revolving Fund (PAHO-RF) vaccine price for 2022] [17, 18]. Furthermore, the earlier HPV vaccination program is initiated, the greater health and cost benefits will be gained [8]. Regarding the HPV vaccine supply and dose restrictions, Chinese scientists reported that 14-year-old girls should be given priority when on-label use of HPV vaccines [17]. If the one-dose schedule of HPV vaccine recommended by the updated WHO position paper is permitted in China, reallocating the second dose from the routine cohorts to add a catch-up vaccination at 20 years of age would be the most efficient strategy, but more local evidence is needed to support the one-dose HPV vaccination in China [17, 18]. The reduced-dose schedules allow to alleviate the supply-related issues and enhance the vaccination uptake, especially for the hard-to-reach population. The rapid R&D of domestic vaccines and the accumulation of local evidence are driving HPV vaccination in China from available to accessible, providing strong support for its national scale-up in the near future.

### Free HPV vaccination triggered by the Healthy City Innovation Pilot Program

In the present China’s market where HPV vaccines are purchased privately, most HPV vaccines are given to the adult women rather than the young girls aged 9–14 years old (vaccination coverage < 1% in 2020). Introducing HPV vaccination into the NIP is essential to reach 90% of girls vaccinated by the age of 15 by 2030. Before the availability of rolling out the HPV vaccination program in China, local governments with adequate resources are encouraged to pilot the vaccination program firstly, to help increase the HPV vaccination rate and to facilitate its full nationwide rollout [19]. In March 2021, the National Health Commission launched the Healthy City Innovation Pilot Program focusing on cervical cancer comprehensive prevention and control, involving the following targets by 2025: having 90% of girls vaccinated by age 15 years, improving screening coverage of the women aged 35–64 (particularly over 70% of women screened by age 35 years and again by age 45 years), and having 90% of women with cervical cancer and precancerous treated [20]. Under such an initiative, several pilot cities with adequate resources have first initiated local government-funded HPV vaccination programs targeted at young adolescents, providing a promising start for free HPV vaccination in China (Additional file 1: Figure S1).

During the two-year implementation period, most of the pilot regions target girls at 13–14 years of age with a delivery strategy from the school-based notification to the designated medical institution- or community health service center-based vaccination. Several flexible funding approaches have been set up for HPV vaccination, including (1) free charge for a certain type of HPV vaccine, or (2) fixed financial compensation whichever types of HPV vaccines, or (3) free charge for one certain type of HPV vaccine combined with fixed financial compensation for other types of HPV vaccines. Meanwhile, all related stakeholders in pilots, including the Health Commission, the departments of Education and Finance, the Centers for Disease Control, the Medical Products Administration, et al., are all engaged in collaboration to promote the HPV vaccination. Up to the end of 2022, all the pilot cities with free or financial compensation policies for the HPV vaccination have achieved vaccination coverage approaching or more than 80% among targeted girls (depending on the city). Such encouraging results may greatly benefit from the joint efforts during the implementation period, including but not limited to the robust implementation by local government, active mobilization and organization of the education department, multisectoral cooperation, and some financial support from the government. Definitely, the effectiveness and sustainability of this innovative mechanism need to be assessed in the long term, in order to inform appropriate expansion pathways of HPV vaccination tailored to China’s enormous population, expansive geography, and strained healthcare system. Pilots for free HPV vaccination in China can be regarded as a ‘learning by doing’ process. Following pilots’ success experience, provincial-level administrative regions (PLADs) such as Guangdong, Hainan, Fujian, Jiangsu, Tibet, Chongqing, and Jiangxi have all rolled out policies on free or subsidised HPV vaccination to all targeted girls at 13 or 14 years of age, and dozens of regions have carried out free or subsidised HPV vaccination programs successively. As of now, regions starting government-funded HPV vaccination have covered 17 PLADs in China (Additional file 1: Figure S1).

### Future challenges and steps forward in scaling up HPV vaccination in China

The Chinese government’s commitment to accelerating the elimination of cervical cancer has provided an unprecedented opportunity for rolling out HPV vaccination in China. In September 2021, the *Program for the Development of Chinese Women (2021*–*2030)* has been indicated to promote HPV vaccination in the pilots [21]. In January 2022, the government showed a positive attitude to gradually launch free HPV vaccinations nationwide, starting in pilot regions [19]. In January 2023, the *Action Plan to Accelerate the Elimination of Cervical Cancer (2022*–*2030)* was put forward by 10 central government departments, involving the plans to continuously promote pilot services that offer girls HPV vaccines [20]. A series of policies and actions have indicated the government’s determination to scale up HPV vaccination in more regions and nationwide. However, several challenges should be considered and addressed timely to support the steps forward.

First, high prices for HPV vaccines are the major concern to make the current pilot program or projects sustainable and scalable. Worldwide, HPV vaccines for Gavi-supported countries are priced at USD 4.6 (bivalent HPV vaccines produced by GSK) and USD 4.5 (tetravalent HPV vaccines produced by Merck) per dose. And 14 middle-income countries (MICs) supplied through UNICEF are also purchasing HPV vaccines at a low price of USD 10.25–14.14 and USD 14.34–26.75 per dose, respectively [22]. In contrast, HPV vaccine pricing in China is significantly higher for both the general public (≥ USD 43 per dose) and government procurement (≥ USD 35 per dose). Notably, the nonavalent HPV vaccine in China costs as high as USD 187 per dose, beyond the reach of most eligible women and the government-funded program. Future studies associated with the optimum price of the vaccine, combined with price negotiation with manufacturers are encouraged. Meanwhile, the approval process of the domestically produced HPV vaccines, especially the new-generation vaccines against more HPV types, should be expedited to force producers to cut prices effectively.

Second, the current supply of HPV vaccine is insufficient to meet the aggregate demand for its national scale in a short term. According to the data derived from the National Bureau of Statistics of China, approximately 80 million doses of HPV vaccines would be needed to cover 90% of the entire cohort of girls aged 9–14. However, according to the manufacturers’ annual report, in 2022, there were about 30 million bivalent vaccine doses, 14 million quadrivalent vaccine doses, and 15 million nonavalent vaccine doses available on the market in China. All the above vaccine doses are inadequate to fulfill the national vaccination program in one year. But hopefully, the success of the ongoing R&D efforts on the HPV vaccine will meet the supply-demand in the near future. Currently, under the supply-constrained scenario, routine vaccination of one year-cohort would be preferred for larger and faster health and economic gains with 14-year-olds being the optimal age for vaccination, and approximately 15 million doses of HPV vaccines are needed annually [17].

Third, more attention should be paid to the girls living in the low-resource areas out of the pilot cities. In China, almost 60% of cervical cancer cases are from rural areas, where cervical cancer awareness is poor and women are less likely to actively seek cervical cancer screening [23]. HPV vaccination should also be prioritized for such group of vulnerable women to protect them from developing cervical cancer and compensate for the absence of effective screening programs. Currently, for regions without adequate resources to support government-funded HPV vaccination, a philanthropic project has been launched to donate HPV vaccines for almost 80,000 young girls in approximately 20 low-resource areas across China (Additional file 1: Figure S1). All the manufacturers with HPV vaccines approved are involved in this project and all the local related stakeholders are engaged to manage the project. Though the donated HPV vaccines can not cover all the 9–14 girls in these areas, the primary target of this campaign is to explore the optimal pathway that is suitable for the low-resource areas to scale up HPV vaccination and benefit more girls. Subsequently, sustainable financial support from the local or national governments is critical and necessary to expand and maintain high vaccination coverage, which also applies to the cities involved in the Healthy City Innovation Pilot Program.

Fourth, there is also an urgent need for local evidence on the efficacy, effectiveness, and safety of the novel HPV vaccination strategies. One-dose schedule of the HPV vaccine has been recommended by the WHO that it would free up vaccine doses to allow more rapid scale-up of vaccination, particularly for the areas facing more severe financial and supply constraints. Emerging evidence from foreign countries has indicated that one dose of vaccine could provide an equivalent level of protection against infection and clinical endpoints as two/three doses for more than 10 years. In China, modelling studies have demonstrated that adopting a one-dose schedule could generate the greatest cost savings and health gains with current constrained supply, but few studies focus on the effectiveness of the one-dose schedule among the Chinese population [17]. In the future, local evidence related to the one-dose strategies of HPV vaccine in China is urgently needed to facilitate its national licensure and help to alleviate the national supply constraints. In addition, the HPV vaccine for males has not been licensed in Chinese mainland. More evidence should be accumulated to support its approval and vaccinating males should be considered when vaccine supply is rising. This gender-neutral HPV vaccination strategy would further expand the health benefits of preventing both cervical cancer and other HPV-related disease.

Moreover, we still need to emphasize the importance of regular cervical screening following the implementation of HPV vaccination. Joint education on vaccination and screening should be carried out in the opportunity of pilot programs. Meanwhile, with the increasing coverage of HPV vaccination, exploring appropriate screening techniques and strategies for the vaccinated population should be a focus in the post-vaccination era. Finally, real-world monitoring through the information systems, such as the cancer registry system and its linkage with individual information on HPV vaccination and screening, is warranted.

## Conclusions

For more than 15 years, Chinese scientists, vaccine manufacturers, healthcare practitioners, policymakers, and other related stakeholders have made a series of efforts around the HPV vaccine development, evaluation, application, and promotion. After the approval of the first HPV vaccine, China has expedited its efforts to catch up with the ten-year delayed gap. The progress achieved so far will greatly prompt the national rollout of the HPV vaccine in the future and speed up cervical cancer elimination in China. At the same time, Chinese HPV vaccines will also alleviate the global HPV vaccine supply constraint to a certain extent, and China’s experience may be a case in point to inform countries that have not yet introduced HPV vaccines. In the next step, China will take full advantage of existing opportunities to make strides towards a transition from a regional to a national HPV vaccination program and to accelerate the achievement of the 90% coverage target of HPV vaccination.

### Supplementary Information

**Additional file 1: Figure S1.** Free or subsidised human papillomavirus vaccination regions in China by April 2023.

## Data Availability

The datasets used and/or analysed during the current study are available from the corresponding author upon reasonable request.

## Abbreviations

HPV: Human papillomavirus
WHO: World Health Organization
NIP: National immunization program
R&D: Research and development
CIN2 +: Cervical intraepithelial neoplasia grade 2 or higher
VIN2 +: Vulvar intraepithelial neoplasia grade 2 or higher
VaIN2 +: Vaginal intraepithelial neoplasia grade 2 or higher
ICER: Incremental cost-effectiveness ratio
GDP: Gross domestic product
PAHO-RF: Pan American Health Organization Revolving Fund
PLADs: Provincial-level administrative regions
MICs: Middle-income countries
UNICEF: United Nations International Children’s Emergency Fund
