# Supplementary material for: Tackling barriers to scale up human papillomavirus vaccination in China: progress and the way forward
Source: Infect Dis Poverty. 2023 Sep 21;12:86. doi: 10.1186/s40249-023-01136-6 (PMC10512493; doi:10.1186/s40249-023-01136-6)
Supplement: Supplementary file 1 — Additional file 1: Figure S1. Free or subsidised human papillomavirus vaccination regions in China by April 2023. [file 40249_2023_1136_MOESM1_ESM.docx]

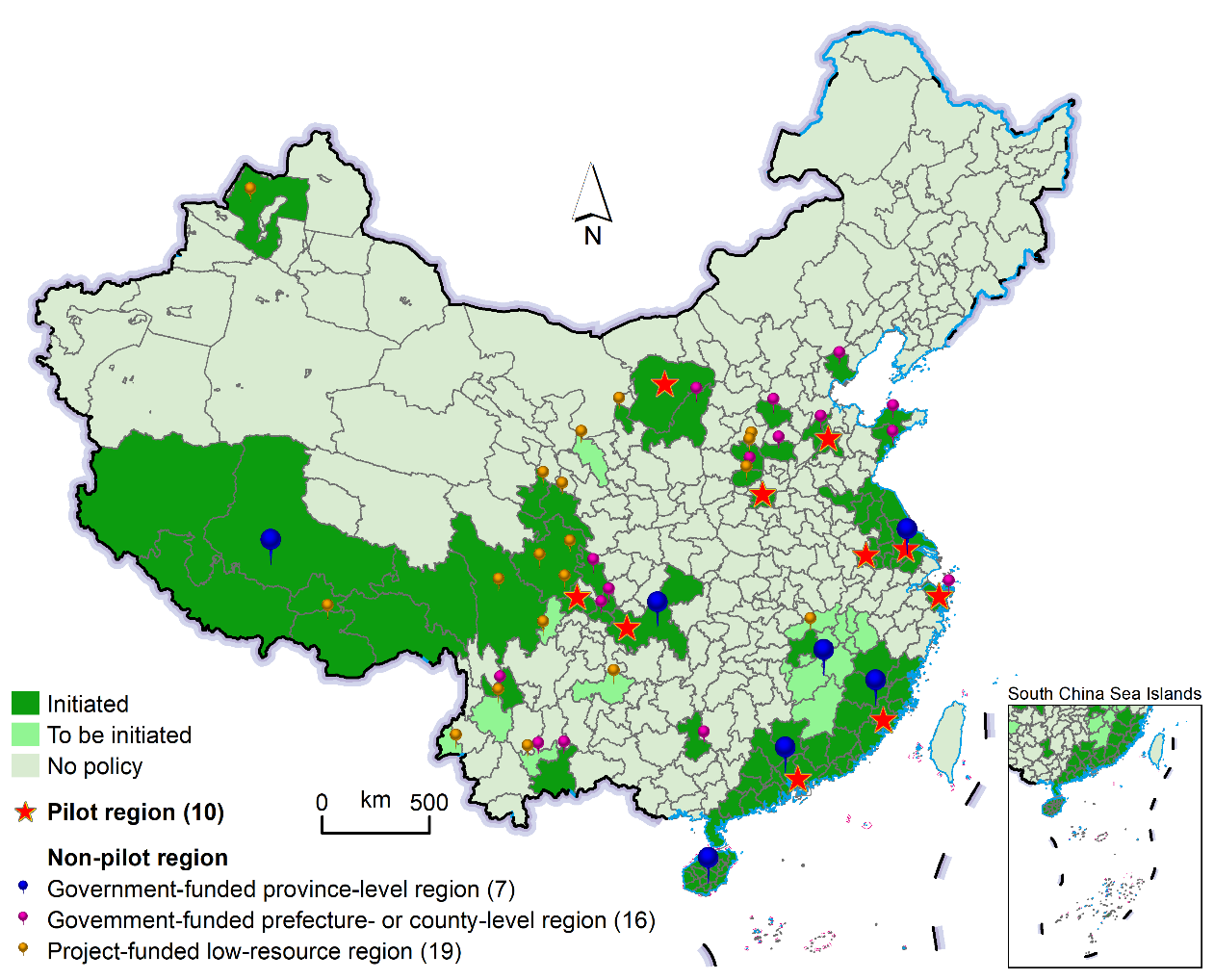


**Figure S1. Free or subsidised human papillomavirus vaccination regions in China by April 2023**

* The base map with an approval number of GS(2022)4299 was downloaded from the designated website (http://bzdt.ch.mnr.gov.cn/) without any modification of the boundaries.
